# Biofortification: how can we exploit plant science and biotechnology to reduce micronutrient deficiencies?

**DOI:** 10.3389/fpls.2013.00429

**Published:** 2013-11-06

**Authors:** Irene Murgia, Laura De Gara, Michael A. Grusak

**Affiliations:** ^1^Department of Biosciences, Università degli Studi di MilanoMilano, Italy; ^2^Centro Integrato di Ricerca, Università Campus Bio-Medico di RomaRoma, Italy; ^3^Department of Pediatrics, USDA-ARS Children's Nutrition Research Center, Baylor College of MedicineHouston, TX, USA

**Keywords:** biofortification, human nutrition, micronutrients, mineral deficiencies, vitamin deficiencies

Throughout the developing world, the long-term consequences of insufficient amounts of essential micronutrients in the human diet can be more devastating than low energy intake. Micronutrients are involved in all aspects of development, growth, and physiology of the human body (including from early embryonic stage), and their deficiencies can cause birth defects, permanent physical and mental impairment, as well as an increased risk of death by infectious and chronic diseases. As plant scientists, we are now facing challenging and urgent goals: how to feed the world's increasing population and how to feed it better. In other words, we need to produce more plant food in sustainable ways and such food should be of the highest nutritional value.

This e-Book aims to provide the most recent advances on plant biofortification for micronutrients as well as a comprehensive overview of the different approaches that can be pursued for producing micronutrient-rich staple plants. We list here some of the major points arising from these papers.

## Vitamins

Vitamins are organic compounds that are required in limited amounts for normal human growth and activity and that cannot be synthesized by the human body. Various genetic engineering approaches for increasing the concentrations of vitamin B_1_, B_6_, C, or D in edible parts of the plant are discussed, together with the possible effects of such increases on plant tolerance to stress.

Acute deficiency in Vitamin B_1_ (thiamin) can result in fatal neurological and cardiovascular disorders. Pourcel et al. (2013) highlight the fact that although the main source of vitamin B_1_ in the diet is plants, there is still a need for enhancing thiamin levels in crop plants because a human diet based mainly on the edible portions of the staple crops, rice, wheat, or maize, is poor in vitamin B_1_. In addition, the refining process for producing white flour and polished rice further reduces their final vitamin B_1_ content. The thiamin biosynthetic pathway is quite complex in plants, utilizing enzymes regulated in a highly complicated fashion, and involving RNA sequences called riboswitches. The most updated pathway scheme achieved in the model plant *Arabidopsis thaliana* is reported in Pourcel et al. (2013), where the various biofortification approaches are discussed in light of the complexity of the pathway itself.

Vitamin B_6_ is involved in different biochemical pathways and in diverse physiological roles in humans that are related to hormones, the immune system, and vascular functions. Adequate vitamin B_6_ intake is beneficial for humans since it reduces the incidence of various health threats, including several multifactorial neurological disorders (Vanderschuren et al., 2013). Differently from vitamin B_1_, *de novo* plant vitamin B_6_ biosynthesis involves only two enzymes, PDX1 and PDX2 (pyridoxin synthesis gene 1 and 2). Vanderschuren et al. (2013) comment on the first successful results in engineering vitamin B_6_ biofortification by overexpression of both enzymes in Arabidopsis. These authors suggest that next steps will be the full understanding of the regulation of vitamin B_6_ biosynthesis, the selection of target food crops, and use of biofortification strategies (including the one applied in Arabidopsis) to such food crops.

Vitamin D maintains and regulates calcium levels in the body. Vitamin D_3_ biosynthesis requires UV-B rays for its photochemical conversion from pro-vitamin D_3_, a process occurring in the human body; therefore, depending on latitude or season, people may be at risk for vitamin D deficiency if sufficient vitamin D is not consumed through supplementation or through a vitamin D-rich diet. Fish are good sources of vitamin D, whereas only small amounts of vitamin D can be found in plants (Jäpelt and Jakobsen, 2013). Nonetheless, Jäpelt and Jakobsen (2013) argue that increased knowledge of vitamin D and of its metabolites in plants, as well as improvements in analytical methods for detection of vitamin D and of its derivatives, can offer new tools for increasing vitamin D content in edible parts of plants. Furthermore, a hypothetical biosynthetic pathway for vitamin D in plants is presented by these authors.

Plant foods are the main dietary source of vitamin C (ascorbic acid; ASC) for humans, for whom severe deficiency causes scurvy and can lead to death. Sub-optimal vitamin C intake, by leading to increased susceptibility to infections and diseases, can represent a health threat for both developing and developed world populations. In the review by Locato et al. (2013), possible plant biofortification strategies for this vitamin are examined, while acknowledging that ASC levels in plants should be considered a complex quantitative trait. Interestingly, ASC levels are tightly linked not only to plant tolerance to stress, but also to sugar biosynthesis and to the regulation of the fluxes of ASC precursors among metabolic pathways (Locato et al., 2013). Transgenic tomato plants overepressing a bacterial pyrophosphatase show increased expression of some genes coding for enzymes involved in the major ASC biosynthetic route in plants (Smirnoff-Wheeler pathway) and such transgenic plants show increased ASC content in fruits, as well as an increase of major sugars (sucrose and glucose) and a decrease in starch content (Osorio et al., 2013).

## Metal micronutrients

Iron (Fe) and zinc (Zn) are two essential metal micronutrients for human health; their deficiencies in the human diet contribute to high rates of mortality in developing countries. Manganese (Mn) deficiency, though less prevalent than Fe and Zn deficiency, can also lead to serious health problems, including birth defects (Bashir et al., 2013). A review of progress in biofortification of rice with such micronutrients is presented in Bashir et al. (2013), where a list of possible bottlenecks for biofortification are also presented, including the avoidance of accumulation of the toxic metal cadmium. The need for better knowledge of seed physiology and morphology is also discussed, in order to ensure successful increases in micronutrient concentrations in the edible parts of plant seeds, especially after the polishing and refining processes are completed. Along these lines, Blair et al. (2013) studied seed coats of common bean seeds to characterize variations in Fe and Zn concentrations in individual lines of a recombinant inbred population. They use these results to identify some of the underlying genetic loci responsible for seed coat accumulation of these metals, which should be useful for future biofortification efforts with bean.

Success in increasing Fe and Zn concentrations in polished seeds of a major rice variety in Myanmar, through a transgenic approach targeting metal transport and accumulation, is presented by Aung et al. (2013). In another study, increases in Fe concentration in a *Japonica* rice variety were also achieved by stacking a set of Fe homeostasis genes (Masuda et al., 2013). Because several iron-related processes were altered (from uptake to chelation and storage), the authors were careful to elevate seed Fe levels without inducing symptoms of Fe deficiency at the whole-plant level. Moreover, they used a marker-free vector that could serve to increase public acceptance of the transformed lines (Masuda et al., 2013). This so-called “push-pull” mechanism of augmenting Fe uptake at one end of the system, along with the storage sink for Fe at the other end of the system (by means of overexpressing Fe transport and storage genes), was investigated in detail in a different transgenic rice line and demonstrated not to interfere with general Fe homeostasis in such plants, relative to non-transgenic controls (Wang et al., 2013). Similar non-target effects of metal-related genes were studied in maize plants expressing a soybean ferritin gene (iron storage protein) in the endosperm (Kanobe et al., 2013). These authors demonstrate that the levels of some metal-related gene transcripts and proteins were indeed changed in the transgenic line, emphasizing the need to take a holistic approach when evaluating transgenic events.

Three different gene families have been described in detail and proposed as possible targets for future Zn or Fe biofortification strategies: the NAC transcription factors (Ricachenevsky et al., 2013a), the metal tolerance proteins (MTP's) (Ricachenevsky et al., 2013b) and the ZIP family of metal transporters (Astudillo et al., 2013). Notably, a candidate gene for increasing Zn concentration in common bean, *PvZIP12*, is proposed in Astudillo et al. (2013) and a model has been proposed for the role of the rice *OsNAC5* gene in senescence and metal re-mobilization (Ricachenevsky et al., 2013a).

## Iodine

Iodine is a non-metal micronutrient that is essential for human health and whose deficiency impairs thyroid functions. When severe deficiency occurs, fetal development can be affected with consequent irreversible brain damage and mental retardation. A possible approach to produce plants biofortified with iodine is to administer exogenous iodine salts to the soil during plant growth. Kato et al. (2013) demonstrate that rice roots possess the ability to reduce IO^−^_3_ to I^−^ and that such reduction is dependent, at least in part, on external iodine conditions. Such results suggest the existence of an iodate reductase in plant roots (Kato et al., 2013). Moreover, Kiferle et al. (2013) demonstrate that tomato fruits can be good targets for iodine fortification, by means of plant irrigation with KIO_3_ during growth.

## Moving forward

The papers presented in this e-Book demonstrate how knowledge in plant metabolism, physiology, and molecular biology can provide approaches for increasing the nutritional value of plant derived foods. The contributions also draw attention to the need for multidisciplinary efforts to cope with the challenges of food security and micronutrient malnutrition. We believe that the information presented in this e-Book will provide several novel ideas and will stimulate new directions amongst researchers in the field of plant biofortification. We also hope that these contributions can serve as a good source of background knowledge to educate and bring new scientists into the field. We are indebted to the many authors who have contributed to this e-Book and for their continued work in this important field of science. We feel privileged to have had the opportunity to oversee and edit this fine group of papers.
